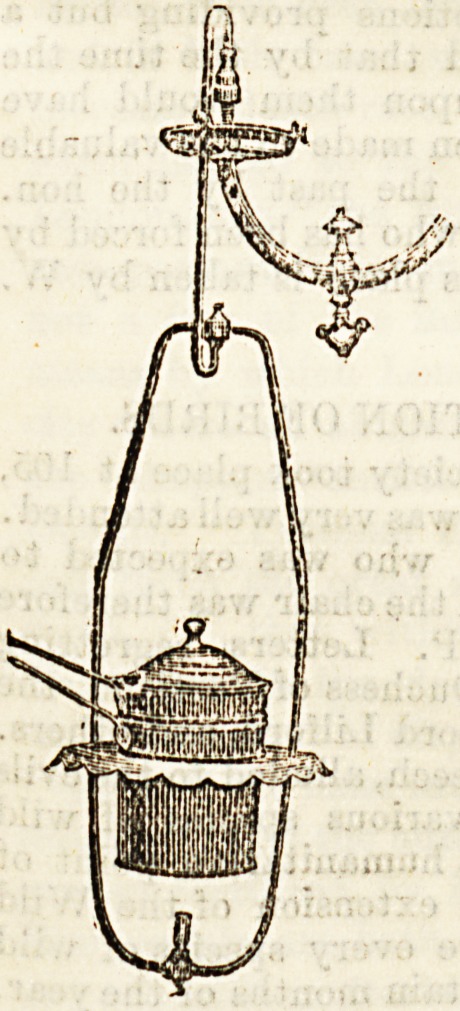# Practical Departments

**Published:** 1894-03-03

**Authors:** 


### PRACTICAL DEPARTMENTS.

### ROYLE'S PATENT GAS ATTACHMENTS.

Pendulous Kettle.

Mr. John Royle, Great Bridgewater Street, Manchester, makes a speciality of such very convenient little appliances as those shown in our illustrations, for attachment to ordinary gas-burners.

The pendulous kettle shown in drawing No. 1 can be hung from any burner, and requires no fixing other than its placing on the small hook. It is perfectly gas-tight, and there is no liability whatever to overturn. Water may thus be boiled in an astonishingly short time, with considerable saving of trouble also. Its price in copper is 5s. 6d.

Food Warmer.

The second drawing shows a small food warmer, also hung pendant from an ordinary gas-burner. Both with this and the kettle one special point is the great convenience of keeping food or water hot with so little trouble by the easy regulation of the gas. Its superiority over a spirit lamp in this respect is very great. The latter are difficult to regulate, even those which are specially made with that intention, and the best of them have an exasperating habit of getting out of order at the critical moment, just when the water is required at boiling point, or milk or beef tea needed at a proper degree of heat. Where gas obtains, therefore, these attachments will be found of great use, and their very moderate price puts them within the reach of most people.

Mr. Royle has many other similar appliances, of which the "Pendulight," a device for suspending or bringing down a lower light from any existing gas-bracket for use as a reading lamp, will, we think, be a real boon to many workers who need a strong light close to a book or work, while its green shade protects the eyes from glare. It is attached or detached in a moment, and is perfectly easy to fix. The list of like conveniences in Mr. Royle's catalogue are really fascinating.

The illustrations are given by kind permission of the inventor.

## Figures and Tables

**Figure f1:**
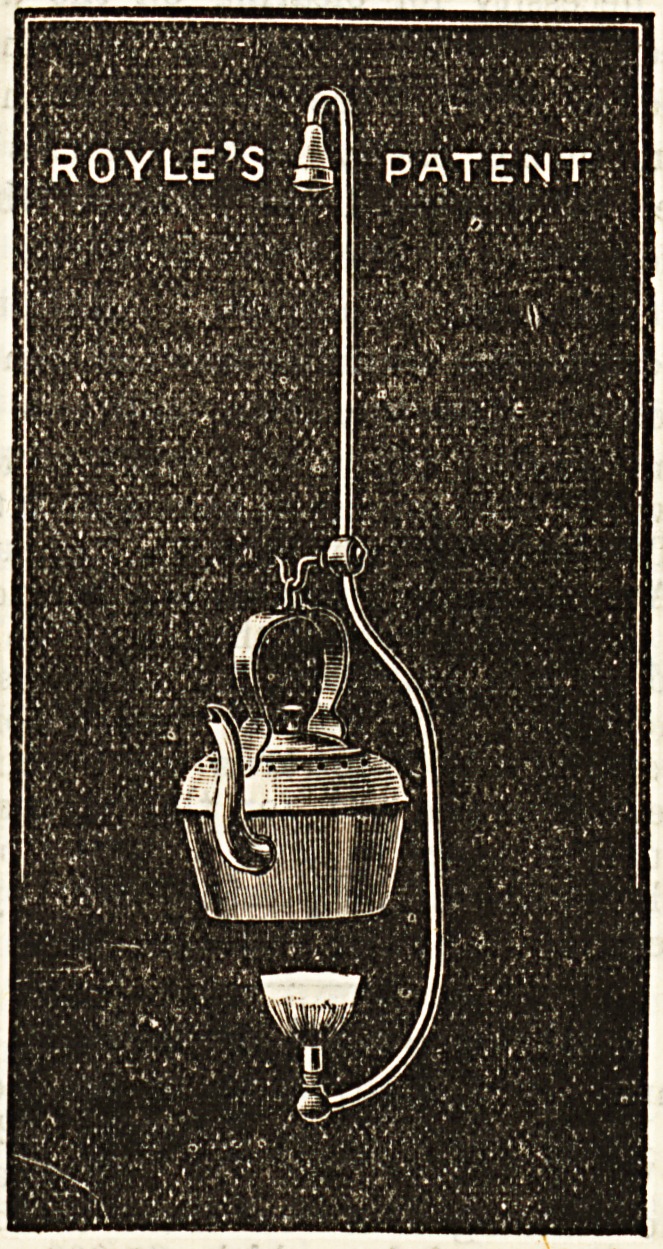


**Figure f2:**